# Mortality and functional outcomes in elderly adults treated surgically by hemiarthroplasty for femoral neck fractures

**DOI:** 10.1177/20503121241307264

**Published:** 2025-01-09

**Authors:** Ankush Ratanpal, Katapadi Ramachandra Kamath, Preetham Raj V Salian, Saiprasad Sarvothama Baliga, Rajendra Annappa, Sayak Banerjee

**Affiliations:** 1 North DMC Medical College and Hindu Rao Hospital, Guru Gobind Singh Indraprastha University, Delhi, India; 2 Department of Orthopaedics, Kasturba Medical College, Mangalore, Manipal Academy of Higher Education, Karnataka, Manipal, India; 3 ESIC Medical College and Hospital and Occupational Disease Center [East Zone], Joka, Kolkata, India

**Keywords:** Mortality after hip fractures, Harris hip score, elderly, Oxford hip scores, hemiarthroplasty

## Abstract

**Background:**

Despite multiple studies, less recent literature and data regarding the mortality associated with hip fractures in the elderly population are available.

**Objectives:**

To assess the mortality data and functional outcomes of patients who underwent cemented and uncemented hemiarthroplasty in femoral neck fractures. To evaluate if preoperative (minimum 2 months) calcium and vitamin D supplement intake in patients affects postoperative mobilization with or without walker support.

**Methods:**

All patients aged 65 and above who underwent hemiarthroplasty for femoral neck fractures in our tertiary care center were included. Postoperative functional outcomes were determined using the Modified Harris Hip score and Oxford Hip score at 3, 6, and 12 months. The mortality of the procedures was assessed at 3, 6, and 12 months. Individuals who took both calcium and vitamin D supplements for at least 2 months before surgery were divided into two groups: those who did not take supplements and those who did.

**Results:**

We studied 110 patients above the age of 65 years. The postoperative mortality rate at 3, 6 months and 1 year postoperatively was found to be 3.6%, 4.7%, and 15.5% respectively. Functional outcomes were assessed at 3, 6, and 12 months postoperatively using modified Harris Hip score and Oxford Hip score and were found to be identical in both cemented and uncemented hemiarthroplasty groups. Patients who took calcium and vitamin D supplements preoperatively (minimum 2 months) could walk without support at the end of 1-year post-surgery.

**Conclusion:**

Early surgery and early mobilization should be the main aim of treatment for femoral neck fractures.

## Introduction

Fractures around the hip are commonly seen in the geriatric population. They are broadly classified into intertrochanteric fracture and femoral neck fracture. It is important to classify the two fractures because their treatments differ greatly from one another.

It has been demonstrated that the morbidity rate for femoral neck fractures in the elderly is 30%. Ramadanov et al.^1^ concluded that an endoprosthetic procedure should be considered in high-risk patients as compared to osteosynthesis (⩾80 years).

In a different study, Ramadanov et al.^2^ concluded that complete hip arthroplasty (hemiarthroplasty) was superior to cannulated screw fixation and dynamic hip screw fixation in terms of significant outcome criteria like Harris Hip score (HHS), EQ-5D (EuroQol 5 Dimension), and reoperation risk.

In elderly patients, there is no scope for nonoperative management. The main goal is early surgery and early mobilization. This is to ensure that the patients don’t develop postoperative complications such as DVT, pneumonia, and bedsores due to prolonged bed rest.

The latest guidelines state that surgical management of femoral neck fractures aims to return a patient to an acceptable functional state as quickly as possible with the least amount of mortality and acceptable quality of life and also avoid revision surgeries.

A retrospective study showed that factors influencing the outcomes of hemiarthroplasty are operation time, patient age, male sex, and operator experience.^3^

Bipolar hemiarthroplasty or total hip replacement are currently accepted surgeries for femoral neck fractures, which enable early postoperative weight-bearing and mobilization.^4^

Patient factors (age, preinjury mobility, comorbidities) must be considered before choosing the surgical approach to get a successful surgical outcome.

However, the risk of prolonged surgery along with the risk involved in cementing should be borne in mind. Few authors support the use of dynamic hip screws or cancellous screws for fracture fixation in the elderly. However, a significantly high nonunion rate is a drawback of internal fixation.

Even though hip fractures have a high mortality rate, the variables that contribute to death following a hip fracture have not been fully researched in a large, worldwide population. Surgeons could treat hip fracture patients more effectively and make better treatment decisions if the variables connected to mortality had a proper association. Cemented bipolar hemiarthroplasty is preferred in the osteoporotic elderly age group.^5^ The complications noted with hemiarthroplasty include persistent hip pain, poor mobility, hip pain and anterior thigh pain, superficial infection, and significant lower limb muscle wasting.^6^

In our study, we assessed the 1-year mortality rates in elderly patients who underwent hemiarthroplasty for femoral neck fracture. We also assessed the difference in mortality in patients who underwent cemented and uncemented hemiarthroplasty. We tried to establish a correlation between the patient’s mobilization with and without walker support and their preoperative calcium and vitamin D intake prior to the trauma.

## Materials and methods

This prospective study was conducted from September 2020 to September 2022 (which included a 1-year follow-up) in the Department of Orthopedics, Kasturba Medical College, Mangalore, and allied hospitals after approval from the Institutional Ethics Committee, Kasturba Medical College, Mangalore (IEC KMC MLR- 09-19/430). The study participants included 110 elderly Indian patients aged 65 years and above who underwent either cemented or uncemented bipolar hemiarthroplasty for femoral neck fractures. Written informed consent was obtained from all study participants. A convenient sampling technique was used.

The following patients were not allowed to participate in the study: those under the age of 65, those with concurrent femur neck fractures and abnormalities or diseases in other lower limb joints, patients with polytrauma, and patients with open fractures, infections, or tumors. Subjects were categorized into two groups based on patients who took preoperative calcium carbonate (1000 mg daily) and vitamin D (2000 IU daily) for 2 months and those who did not.

The correlation between the patient’s mobilization with and without walker support and their preoperative calcium and vitamin D intake previous to the trauma was done.

The preoperative assessment of study participants was done methodically using clinical and radiological examination. Plain radiographs were used to confirm the diagnosis of femoral neck fractures. All patients were operated by surgeons trained in arthroplasty.

Postoperative clinical assessment was done using the Modified HHS and Oxford Hip score (OHS) at 3, 6, and 12 months.^7,8^

The postoperative radiological outcomes were studied using postoperative radiographs of the pelvis including both hips, obtained at 3, 6, and 12-month follow-ups. The patient mortality was recorded at 3, 6, and 12 months.

## Statistical analysis

Data were entered into Microsoft Excel and statistical analysis was carried out in IBM SPSS (statistical package for social sciences) software version 17.0. Qualitative variables were presented as frequency and percentages. Quantitative variables were presented as mean (standard deviation) or median (range) depending upon the distribution of data. Bar diagrams and pie charts were used for the graphical representation of data. The comparison of socioeconomic status and type of surgery with mortality was assessed using the Chi-squared test. Similarly, the effect of calcium/vitamin D intake on walking with or without support was assessed using the Chi-squared test. Type of surgery and HHS categories or OHS categories were compared using a Chi-squared test and a *p*-value of less than 0.05 was considered as statistically significant.

## Result

The patients who underwent surgery for a femoral neck fracture were included in the study. The total number of participants was 110. Most of the participants were between the ages of 65 and 69. The majority of the patients were females (66%), 81.8% of patients had co-morbidities and 18.2% had no comorbid illness. The demographic details have been added in (Table 1). Postoperative infection of the surgical site was noted in only one patient. Only surgical site infection was considered in this study. The percentage of patients who took preoperative calcium and vitamin D supplementation for a minimum of 2 months was 87.3%. Out of the 110 patients included in the study, 39% underwent bipolar cemented hemiarthroplasty, and 61% individuals underwent bipolar uncemented hemiarthroplasty.

**Table 1.** Demographic variables.

Demographic variables	Frequency	(%)
1. Age		
65–69	63	57.3
70–74	21	19.1
75–79	11	8.2
80–84	9	5.4
85 and above	6	10
Total	110	100
2. Gender		
Male	37	33.6
Female	73	66.4
Total	110	100
3. Comorbidities		
Patients with comorbidities	90	81.8
(a) Diabetes mellitus type 2	37	
(b) Hypertension	40	
(c) Coronary artery disease	4	
(d) Respiratory disease	3	
(e) Human immunodeficiency virus	1	
(f) Hypothyroidism	2	
(g) Cerebrovascular accident	2	
(h) Chronic kidney disease	1	
Patients without comorbidities	20	18.2
Total	110	100
4. Cemented hemiarthroplasty	Excellent score	Good score
(A) HHS at the 12th month	72.70%	13.60%
(B) OHS at 12th month	59.10%	13.6
(C) Mortality at 1 year	12%	
5. Uncemented hemiarthroplasty		
(A) HHS at the 12th month	4.30%	61.40%
(B) OHS at 12th month	4.30%	61.40%
(C) Mortality at 1 year	15.70%	

At the end of 3 months, the majority of individuals had fair scores (65.1%), at the end of 6 months the majority of individuals again, had fair scores (50.5%), and at the end of 12 months, the majority of individuals had good scores (60.2%) with 6.5% individuals having excellent scores. Mortality rate at 3 months, 6 months, and 1 year was noted to be 3.6%, 4.7%, and 7.9%, respectively. The overall mortality rate was reported to be 15.5%. Out of the total 93 patients alive at the end of 1 year, 57 patients could walk without support and reached their pre-fall status. At the end of 1-year, identical mortality data were noted in both cemented and uncemented groups of 12% and 15.6%, respectively.

**Figure 1.**
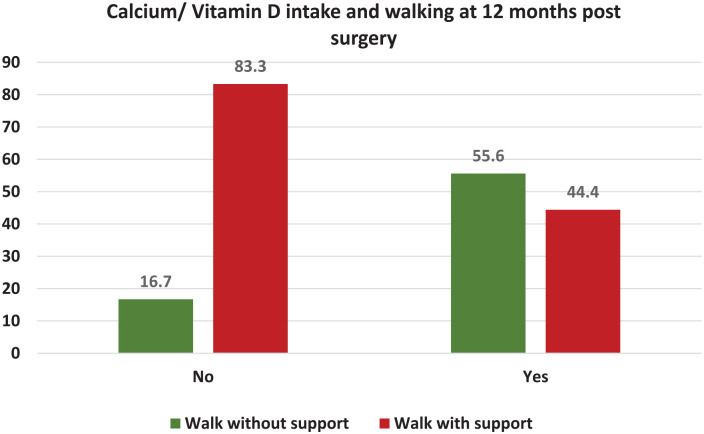
Correlation between walking and vitamin D and calcium daily supplementation.

A significant relation was found between calcium and vitamin D intake for 2 months preoperatively and walking without the use of walking aids at the end of 1 year (*p*-value = 0.012). The participants who took preoperative calcium and vitamin D supplementation (55.6%) were able to walk without support at the end of 1 year postsurgery (Figure 1).

**Figure 2.**
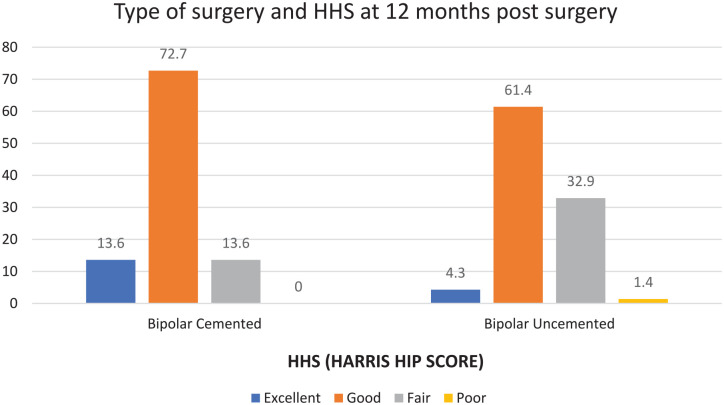
HHS for cemented and uncemented groups.

Comparative analysis of modified HHS at the end of 3, 6, and 12 months postoperatively showed at the end of 3 months, the majority of individuals had fair scores (58.5%), at the end of 6 months, the majority of individuals had good scores (52.5%), and at the end of 12 months, majority individuals had good score (63.4%) with 6.5% individuals having excellent scores (Figure 2).

The modified HHS at 3, 6, and 12 months in both cemented and uncemented groups were compared. At the end of 3 months postoperatively, the majority of individuals in the cemented group had good scores, compared to the uncemented group, in which the majority of individuals had fair scores.

**Figure 3.**
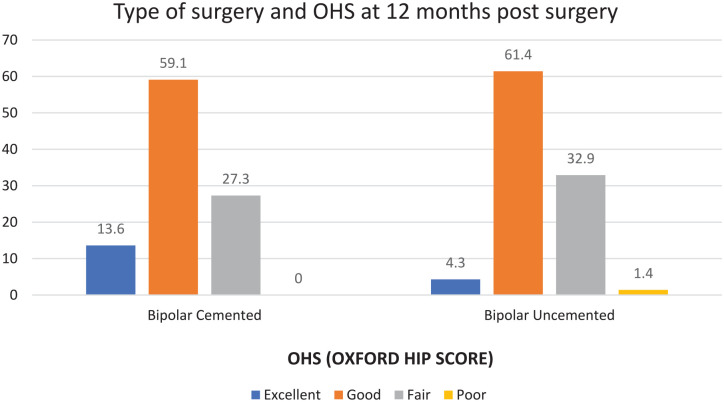
OHS in cemented and uncemented groups at 12 months.

At the end of 6 months postoperatively, the majority of individuals in the cemented group had good scores (72.7%) as compared to the uncemented group in which there was an equitable distribution noted between fair scores (48.7%) and good scores (47.4%). At the end of 12 months postoperatively, the majority of individuals in the cemented group had good scores (72.7%) with 13.6% having excellent scores as compared to the uncemented group in which 61.4% individuals had good scores and 4.3% had excellent scores. At the end of 12 months postoperatively, the majority of individuals in the cemented group had good OHS scores (59.1%) with 13.6% having excellent scores as compared to the uncemented group in which 61.4% of individuals had good scores and 4.3% had excellent scores (Figure 3). At the end of 3 months postoperatively, the majority of individuals in the cemented and the uncemented group had fair scores.

In contrast to the uncemented group, where equal distribution of fair scores (52.6%) and good scores (42.3%) was observed, the majority of people in the cemented group had good OHS scores at the 6-month postoperative mark (59.1%).

At the end of 12 months postoperatively, the majority of individuals in the cemented group had good scores (59.1%) with 13.6% having excellent scores as compared to the uncemented group in which 61.4% individuals had good scores and 4.3% had excellent scores.

## Discussion

The success of hemiarthroplasty is determined not only by surgical technique but also by patient factors. The patient’s age, comorbidities, and compliance with treatment play an important role in successful postoperative rehabilitation.

As mentioned previously the mortality rate for hip fractures is around 30% and studies have been conducted to determine the factors responsible and how to manage them.

In our prospective study with one-year follow-up, we had two patients with immediate postoperative mortality in the cemented group on post-op day 1 and post-op day 7. However, the one-year mortality was almost identical in both cemented (12%) and uncemented groups (15.7%).

In our study, we found that only one person without a comorbid disease had postoperative death, while the majority of people with preexisting comorbidities died after surgery. Only two of the seven people who died from CVA (cerebrovascular accident) had a prior history of the condition, and five of them were hypertensive and diabetic.

Three of the seventeen patients who died overall received DVT (deep vein thrombosis) prophylaxis for 35 days following surgery. Additionally, two people died at POD (postoperative day) 1 and 7, respectively, and were unable to mobilize in the immediate postoperative period. On the second postoperative day, the remaining patients were moved and allowed to bear full weight.

Of the 110 participants in the study, one experienced a refracture on the same side fracture 4 months after fixation, and two had a history of femoral neck fractures on the opposite side with prior hemiarthroplasty. A single patient complained of excruciating back discomfort, which started 1 year before surgery.

After a year, the overall mortality rate was 15.5%, with 15.6% in the uncemented group and 12% in the cemented group. Quality of life at the end of 1 year was identical in all individuals irrespective of the type of surgery. In our study, the mortality data at 3 months, 6 months, and 1 year was noted to be 3.6%, 4.7%, and 15.5%, respectively. In our study, 57 out of a total of 93 patients were able to walk without support and were able to reach their prefall status. In our study, we found a positive relationship between patients who consumed calcium and vitamin D supplements for a minimum period of 2 months preoperatively, and those who were able to walk without support at the end of 1 year.

Patients with preoperative calcium and vitamin D intake were able to walk without support at the end of 1-year postsurgery. At the end of a year, a study by Fenelon et al.^9^ in Ireland found no difference in mortality between patients who had hemiarthroplasty with cement or without cement.

A study by Fenelon et al.^9^ showed that after 6 months, the cemented group (72.7% of the individuals had good modified HHS) performed better than the uncemented group (47.4% had good modified HHS). But neither the cause of mortality nor the use of calcium and vitamin D were covered in this study.

Jensen et al.^10^ in Denmark found the majority of individuals belonging to the female sex, similar to our study. However, they found the mean age to be 77 years contrary to the 70.4 years found in our study. Death rates were 17%, 21%, and 27% at the end of the study’s first year.

Ponraj et al.^11^ in Tamil Nadu, India, the mean age was found to be 64 years with a female-to-male ratio of 1.7:1. In our study, the female-to-male ratio was found to be 1.97:1. He also compared the functional outcome using the HHS between the cemented and the uncemented hemiarthroplasty group. They found that the scores were identical in both the groups at the end of 1 year. Similar findings were found in our study; however, Cecilia et al concluded that at the end of 1 year, 32 out of a total of surviving 70 patients who underwent hemiarthroplasty were able to mobilize without walking aids and were able to reach their prefall status.

We studied the causes of mortality during the 1-year postoperative follow-up and concluded that cerebrovascular accidents (7 of 17 deaths) constitute major causes of mortality followed by cardiovascular (5 of 17 deaths), and respiratory (4 of 17 deaths) causes. In a study by Rogmark et al.,^12^ respiratory disease was found to be the leading cause of death, followed by cerebrovascular accident and congestive heart failure. The results showed that, after a year, the death rates from cemented and uncemented bipolar hemiarthroplasty groups were the same, at 24.5% for the cemented group and 26.4% for the uncemented group.

The limitations of our study were a short postoperative follow-up period and a small sample size. We did not perform a power analysis for sample size collection. We would recommend future studies of a longer follow-up duration and larger sample size be conducted with the inclusion of other hip fractures in the elderly.

## Conclusion

Elderly patients with femoral neck fractures treated with a bipolar prosthesis experienced a high rate of recovery and a satisfactory outcome. One year postsurgery, the patients who took calcium and vitamin D supplements preoperatively were able to walk without assistance. Osteoporosis, multiple comorbidities, back pain, and osteoarthritis in the knees are among the morbid problems that keep the person from reaching their prefall level of mobility. Early surgery and early mobilization should be the main aim of treatment in femoral neck fractures.
